# Disorders of Upper Limb Movements in Ataxia-Telangiectasia

**DOI:** 10.1371/journal.pone.0067042

**Published:** 2013-06-27

**Authors:** Aasef G. Shaikh, David S. Zee, Allen S. Mandir, Howard M. Lederman, Thomas O. Crawford

**Affiliations:** 1 Department of Neurology, Case Western Reserve University, Cleveland, Ohio, United States of America; 2 Department of Neurology, The Johns Hopkins University, Baltimore, Maryland, United States of America; 3 Department of Neurology, Georgetown University, Washington D.C., United States of America; 4 Department of Pediatrics, The Johns Hopkins University, Baltimore, Maryland, United States of America; 5 Department of Medicine, The Johns Hopkins University, Baltimore, Maryland, United States of America; University Medical Center Groningen UMCG, The Netherlands

## Abstract

Ataxia-telangiectasia is known for cerebellar degeneration, but clinical descriptions of abnormal tone, posture, and movements suggest involvement of the network between cerebellum and basal ganglia. We quantitatively assessed the nature of upper-limb movement disorders in ataxia-telangiectasia. We used a three-axis accelerometer to assess the natural history and severity of abnormal upper-limb movements in 80 ataxia-telangiectasia and 19 healthy subjects. Recordings were made during goal-directed movements of upper limb (kinetic task), while arms were outstretched (postural task), and at rest. Almost all ataxia-telangiectasia subjects (79/80) had abnormal involuntary movements, such as rhythmic oscillations (tremor), slow drifts (dystonia or athetosis), and isolated rapid movements (dystonic jerks or myoclonus). All patients with involuntary movements had both kinetic and postural tremor, while 48 (61%) also had resting tremor. The tremor was present in transient episodes lasting several seconds during two-minute recording sessions of all three conditions. Percent time during which episodic tremor was present was greater for postural and kinetic tasks compared to rest. Resting tremor had higher frequency but smaller amplitude than postural and kinetic tremor. Rapid non-rhythmic movements were minimal during rest, but were triggered during sustained arm postures and goal directed arm movements suggesting they are best considered a form of dystonic jerks or action myoclonus. Advancing age did not correlate with the severity of involuntary limb movements. Abnormal upper-limb movements in ataxia-telangiectasia feature classic cerebellar impairment, but also suggest involvement of the network between the cerebellum and basal ganglia.

## Introduction

Ataxia-telangiectasia (A–T) is a recessively inherited multi-system disorder with prominent neurodegeneration, immunodeficiency, radiosensitivity, and enhanced risk for lymphoreticular malignancy. Pathological studies in A–T have focused predominantly upon abnormalities of the cerebellar cortex, leading early investigators to identify the disorder as a form of cerebellar ataxia, hence its iconic name [1]. Nonetheless, motor abnormalities of putative extra-cerebellar origin in individuals with A–T have been noted from the earliest case reports [2], [3]. Some of these features, including resting tremor, suggest dysfunction of the basal ganglia. The pathophysiologic basis of other non-rhythmic adventitious movements, such as dystonia and myoclonus could be cerebellar or extra-cerebellar. This uncertainty about the pathophysiology of abnormal motor control is matched by a paucity of literature that quantifies movement disorders in A–T. Measurement of mixed, hyperkinetic movement disorders in A–T might help us to understand their phenomenology, anatomical substrate, and may assist in development of therapeutics. For example, patients with disorders prominently affecting the basal ganglia benefit from a different treatment strategy than those with substantial cerebellar involvement.

We evaluated abnormal limb movements in A–T with three intents: (1) to quantify the severity of tremor and non-rhythmic adventitious movements, (2) by a cross-sectional approach to determine whether, and to what extent, these movement disorders correlate with increasing age, and (3) to determine whether these movement disorders have specific features that support either a pure cerebellar etiology or are instead better explained by mixed cerebellar and extra-cerebellar correlates.

## Methods

### Ethics Statement

The study was approved by The Johns Hopkins Institutional Review Board (NA-00014314). The written informed consent was obtained for the experiment protocol. In instances when participants were minors or children, the written informed consent was obtained from their guardians. The consent form was also approved by the Johns Hopkins Institutional review board. The investigation was conducted according to the principles expressed in the Declaration of Helsinki. The guardian of the subject of the video recording (as the subject was a minor) had given written informed consent, as outlined in the PLOS consent form, to publication of this video. Subjects with A–T were recruited from The Johns Hopkins A–T Clinical Center (ATCC). All subjects met the previously defined criteria for the diagnosis of A–T [4]. Approximately 350 patients with A–T have been assessed at the Ataxia-Telangiectasia Clinical Center (ATCC) at Johns Hopkins. All patients visiting the ATCC of appropriate age and sufficient maturity to cooperate with the study were invited to participate. Subjects taking medications targeting abnormal movement or tone were excluded. Healthy subjects with no clinical evidence of central or peripheral nervous system disorders were recruited for comparison.

A light-weight three-axis accelerometer, mounted on to the dorsum of the middle phalange of the index finger, was used to record movements. Subjects held their arms outstretched against gravity (postural task), comfortably rested their forearms and hands in their lap (rest), and performed slow back-and-forth finger-to-nose movements (kinetic task). Limb movement during each task was recorded for 120 seconds. The signal was recorded at 100 Hz sampling rate.

**Figure 1:**
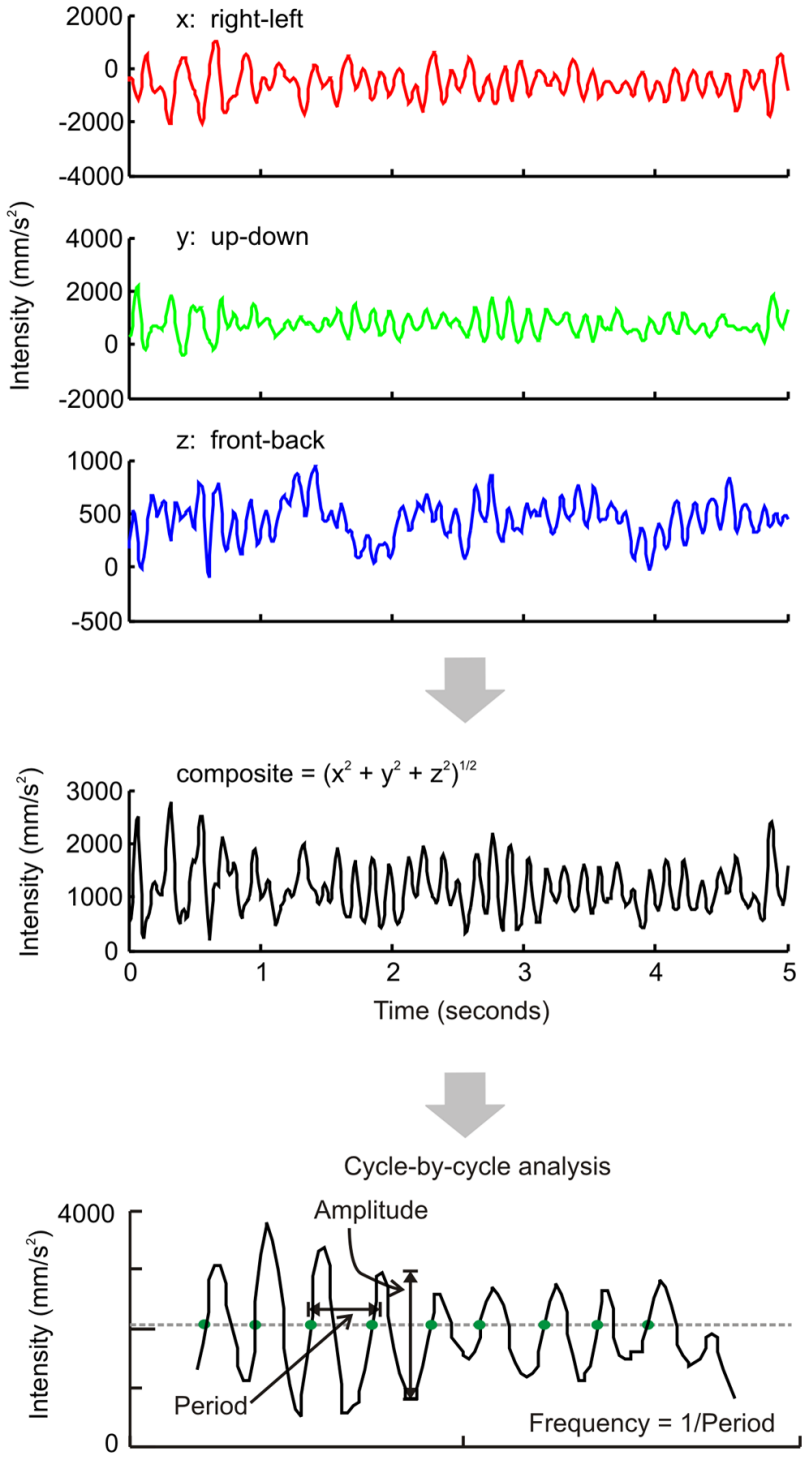
Outline of data analysis. (A) Limb acceleration is plotted on ordinate while corresponding time is plotted on abscissa. Each color depict one axis, black trace depicts sum of intensity vector depicting its composite value. Further analysis was performed on composite vectors. (B) This panel depicts algorithm to measure frequency of oscillations. The acceleration is first aligned to zero, then x-coordinate of the intercept between composite vector and zero-line (grey dashed line) is acquired (“zero-crossing”, red circles in the figure). Alternate value of such “zero-crossing” for acceleration value moving from low to high numbers are used to assess period. Latter is the time difference between two corresponding zero-crossings. Inverse of the period is frequency of given cycle. Population of single-cycle frequency is used to compute weighted mean, and standard deviation (irregularity) of the tremor frequency during given condition.

Each axis of a three-axis accelerometer will record inertial acceleration, and if it changes in orientation an associated change in acceleration attributable to gravity [5]. An accelerometer cannot itself distinguish between gravity and inertia. Each axis will record the modulation of gravito-itertial acceleration within its dedicated axis (i.e. the individual x, y, and z-axes, Figure 1A). The data from each of the three channels were combined into a composite vector (Figure 1A). Although accelerometry cannot distinguish change in gravity from the inertial acceleration, it did not affect our goal to compare within individual patients the quantitative burden of rhythmic movements with non-rhythmic counterparts that contain components of a similar frequency.

### Data Analysis

Accelerometry is inherently associated with high-frequency sampling noise. A moving average algorithm was used to filter the sampling noise. Cycle-by-cycle analysis was performed on smoothed three-dimensional composite vectors. The following steps were followed to define an individual cycle: (1) Data was normalized and de-trended data with the mean intensity (i.e., normalized intensity = actual intensity – mean intensity). This permits data to be realigned along the abscissa such that the peaks of the cycles remained positive and the troughs negative. (2) The ‘x’ co-ordinate of the intersection of the data trace with the abscissa (moving from the negative value to the positive value) was recorded. The x-coordinate of the first data point that crosses the abscissa marked the beginning and the subsequent data point marked the end of the given cycle. 3) With this definition of cycle, its width was computed. The inverse of the cycle width yields the cycle frequency and the difference between the peak and trough measures the cycle intensity. In this technique, the “intensity” of movement was measured in the form of acceleration. (4) We were then able to assess cycle-by-cycle variation of tremor frequency (i.e. standard deviation (SD) of the frequency) within the defined tremor intervals. Figure 1B depicts the outline of cycle-by-cycle analysis to measure tremor frequency. The standard deviation of tremor frequency provides a quantitative estimate of tremor irregularity [6]. Investigations of rhythmic and regular oscillations such as essential tremor find that median frequency measured with the cycle-by-cycle analysis tightly correlates with the dominant spectral frequency [7]. In such regular oscillations the area under the power-spectrum curve is confined to a narrow frequency range. In contrast, the irregular and mixed frequency oscillations of A–T yield a power spectrum span across a broad range of frequencies with multiple spectral peaks, often with comparable power [7]. This thus poses a challenge to identification of an actual dominant frequency. A cycle-by-cycle analysis best addresses this difficulty by computing the mean oscillation frequency and weighing its value with the oscillation amplitude. Hence weighted mean frequency better represents the actual frequency trend. We computed weighted mean frequency for all patients during each movement task. The population of the weighted mean frequency was used to perform further analysis as described in the results section.

The transient episodes of tremor were determined according to the criteria defining tremor as a *rhythmic* movement with a relatively fixed frequency over the determined interval. In this study intervals of tremor were defined as those times when the frequency of three or more consecutive cycles varied by 2 Hz or less.

## Results

**Figure 2:**
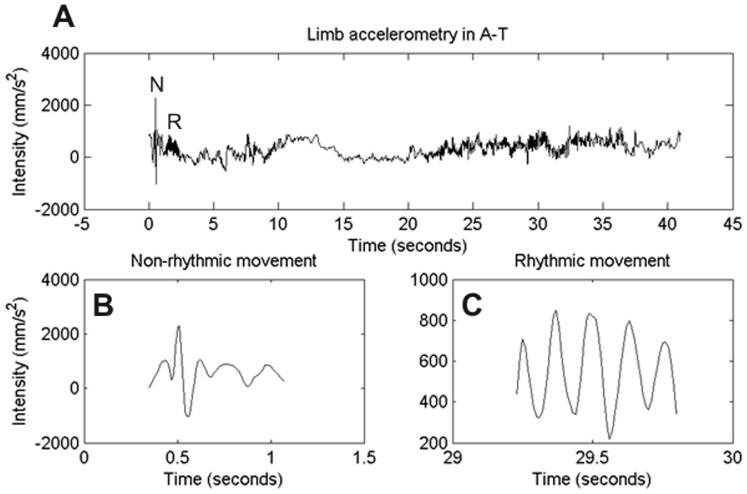
Accelerometry from one A–T subject during postural condition. Composite vector of limb acceleration is plotted on Y-axis, while corresponding time plotted on the X-axis. (A) The trace depicts 40 second epoch of mixed rhythmic and non-rhythmic limb movements. (B) This panel depicts non-rhythmic movement marked as ‘N’ in panel ‘A’. (C) Expanded version of the region that is marked as ‘R’, rhythmic movement, in panel ‘A’.

Accelerometer recordings were performed on 80 A–T subjects (36 males and 44 females, age range: 5 to 34 years, mean: 13.8 years, median: 12.1 years) and 19 age-matched control subjects. Figure 1 illustrates an example of one A–T subject holding the arms outstretched against gravity (postural task). Intensity of limb movement (y-axis) is plotted against time (x-axis). The trace qualitatively shows slowly modulated drifts but also two forms of superimposed fast movements (Figure 2A). One is non-rhythmic, isolated, and appeared intermittently (Figure 2B, expanded from region of Figure 2A marked as ‘N’) and the other is rhythmic in clusters and appeared episodically (Figure 2C, expanded from region of Figure 2A marked as ‘R’). The non-rhythmic fast movement qualitatively resembled a dystonic jerk or myoclonus, while the episodic rhythmic movements had the quality of tremor. The video clip S1 depicts an example of both types of movements in a patient with A–T.

### Rhythmic Movements - Tremor

Rhythmic movements that fulfilled the definition of tremor were observed when the A–T subjects were at rest (resting tremor), with outstretched upper limbs holding them in a sustained position against the constant force of gravity (postural tremor), and while reaching between the examiner’s finger and subject’s own nose or chin (kinetic tremor). Postural and kinetic tremor was present in 79 of 80 A–T subjects, while a resting tremor was also present in 48 (61%). The intensity of postural and kinetic tremor was higher compared to resting tremor in all A–T subjects. None of the control subjects had recordable postural, resting, or kinetic tremor.

**Figure 3:**
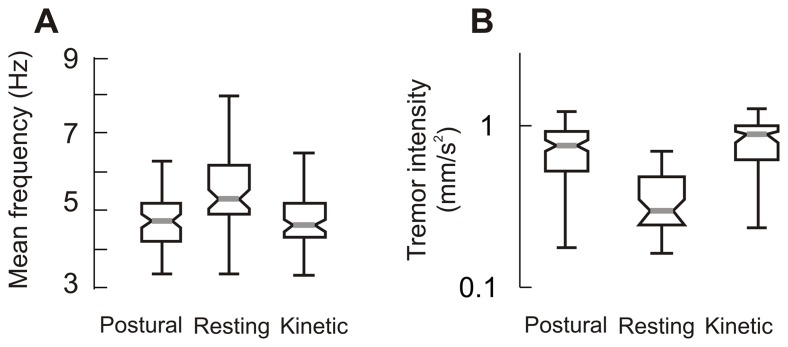
Summary of the frequency and intensity of the postural, resting and kinetic tremor from 79 A–T subjects. Frequency (A) and intensity (B) is plotted on the y-axis. Each box-whisker plot depicts one condition. Grey horizontal line depicts median value, notches correspond to 95% confidence interval, length of box depict quartile values, and whiskers depict range. Panel ‘A’ compares the frequency, while panel ‘B’ compares the intensity.

Box and whisker plots in Figure 3A summarize the frequency of postural, resting and kinetic tremor from 79 A–T subjects. The grey line in the center of each box represents the median frequency and the notch borders the 95% confidence interval of the median. An overlap of notches of two plots suggests an absence of significant difference between the median of the corresponding populations (i.e. ANOVA, p>0.05). The frequency of the postural and kinetic tremor ranged between 3 to 6 Hz. Median frequency of postural tremor was 4.7 Hz and that of kinetic tremor was 4.6 Hz. Resting tremor manifested across a broader range of frequencies, between 3 to 8 Hz (median: 5.3 Hz). The frequencies of the postural and kinetic tremor were similar to one another (One-way ANOVA, p>0.05) but lower than that of resting tremor (One-way ANOVA, p<0.05). In order to compare the severity of rhythmic movements amongst different subjects and experiment conditions, we computed the intensity of these movements per unit cycle (i.e., normalized intensity). Normalized median intensities of the postural, resting, and kinetic tremor were 0.69, 0.14, and 0.71 mm/s^2^, respectively (Figure 3B). The median intensities of postural and kinetic tremors were not significantly different from each other (One-way ANOVA, p>0.05), but significantly larger than resting tremor (One-way ANOVA, p<0.05).

The higher resting tremor frequency compared to that of kinetic and postural tremor is attributable either to lower intensity or reflects a central pathophysiology that is different from that underlying the kinetic and postural tremor [8]. These two possibilities can be distinguished by investigating the difference between resting and kinetic or postural tremor frequencies while controlling for the intensity. Analysis of covariance (ANCOVA), which takes the population mean of the tremor intensity into account while analyzing the difference between the tremor frequencies, suggested a significant difference in frequencies of resting tremor as compared to postural and kinetic tremor (ANCOVA, p<0.05, F = 17.16). Another issue is a possible relationship of low intensity and high frequency resting tremor in A–T to that of physiologic tremor, which in normal conditions is irregular, low amplitude, and of high frequency. If the resting tremor of A–T is related to an exaggerated physiologic tremor it should be relatively irregular (more cycle-by-cycle irregularity) as compared to postural and kinetic tremor [9], [10]. In subjects with A–T, however, the irregularity in kinetic, postural, and resting tremor were not significantly different (Figure, 4A; One-way ANOVA, p>0.05).

**Figure 4:**
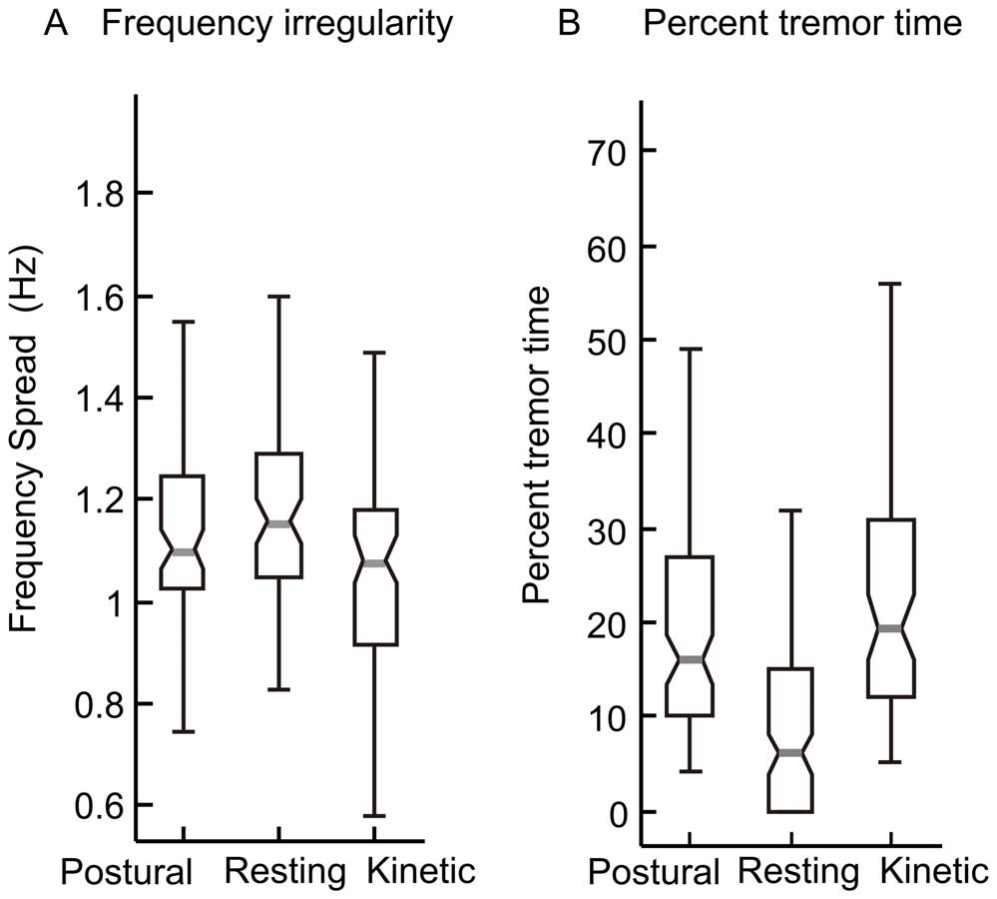
Comparison of the frequency irregularity and percent tremor time during postural, resting, and kinetic tremor in 79 A–T subjects. Each box-whisker plot depicts one condition. Grey horizontal line depicts median frequency spread (A) or percent time (B), notches correspond to 95% confidence interval, box-lengths depict quartile values, and whiskers depict range.

Given the episodic nature of the tremor, we identified the fraction of total recorded time during which tremor was present (percent tremor time). The median values of postural, resting and kinetic percent tremor time were 16%, 6%, and 19.5% respectively (Figure 4B). Percent time for resting tremor was significantly less than postural and kinetic tremor (One-way ANOVA, p<0.05). There was no significant difference between postural and kinetic tremor percent time (One-way ANOVA, p>0.05).

These quantitative differences between postural, kinetic, and resting tremor suggest that postural (or kinetic) and resting tremor all have central etiology, but possibly discrete correlates.

### Non-rhythmic Movements

Rapid non-rhythmic adventitious movements were present in all A–T subjects, but were not identified in any of the controls. These movements were of two types – slow movements appearing like athetosis or dystonic drifts, and fast movements resembling myoclonus or dystonic jerks.

**Figure 5:**
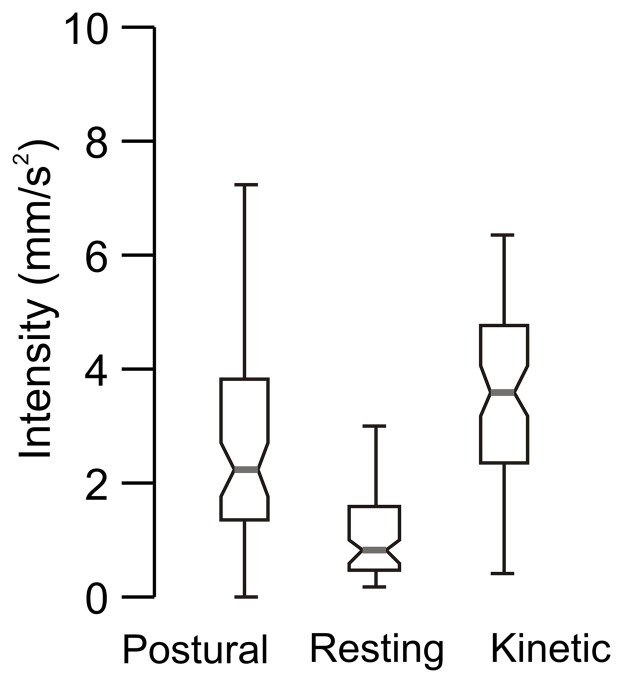
Comparison of the median intensities of non-tremor, rapid, and adventitious movements in 79 A–T subjects. Each box-whisker plot depicts one condition. Grey horizontal line depicts median value, notches correspond to 95% confidence interval, box lengths depict quartile values, and whiskers depict range.

Rapid myoclonic or dystonic movements were also present during rest but increased with activity. Accelerometry allowed quantitative measurement of the rapid, non-rhythmic adventitious movements. In order to compare the severity of these movements amongst different subjects and conditions, we normalized the intensity of these movements per unit time. During resting condition, non-rhythmic movements had normalized median intensity of 1.1 mm/s^2^ (central grey horizontal lines in box and whisker plots in Figure 5). The median intensity of the non-rhythmic movements increased significantly to 3.2 mm/s^2^ during the postural condition (One-way ANOVA, p<0.05) and further to 4.5 mm/s^2^ in kinetic condition (One-way ANOVA, p<0.05).

The quantitative characteristics of non-tremor movements suggest possible role of limb muscle activation to increase their incidence. The results point to the impairment of neural networks between the cerebellum and basal ganglia.

### The Relationship of Tremor and Non-rhythmic Movements to Age

**Figure 6:**
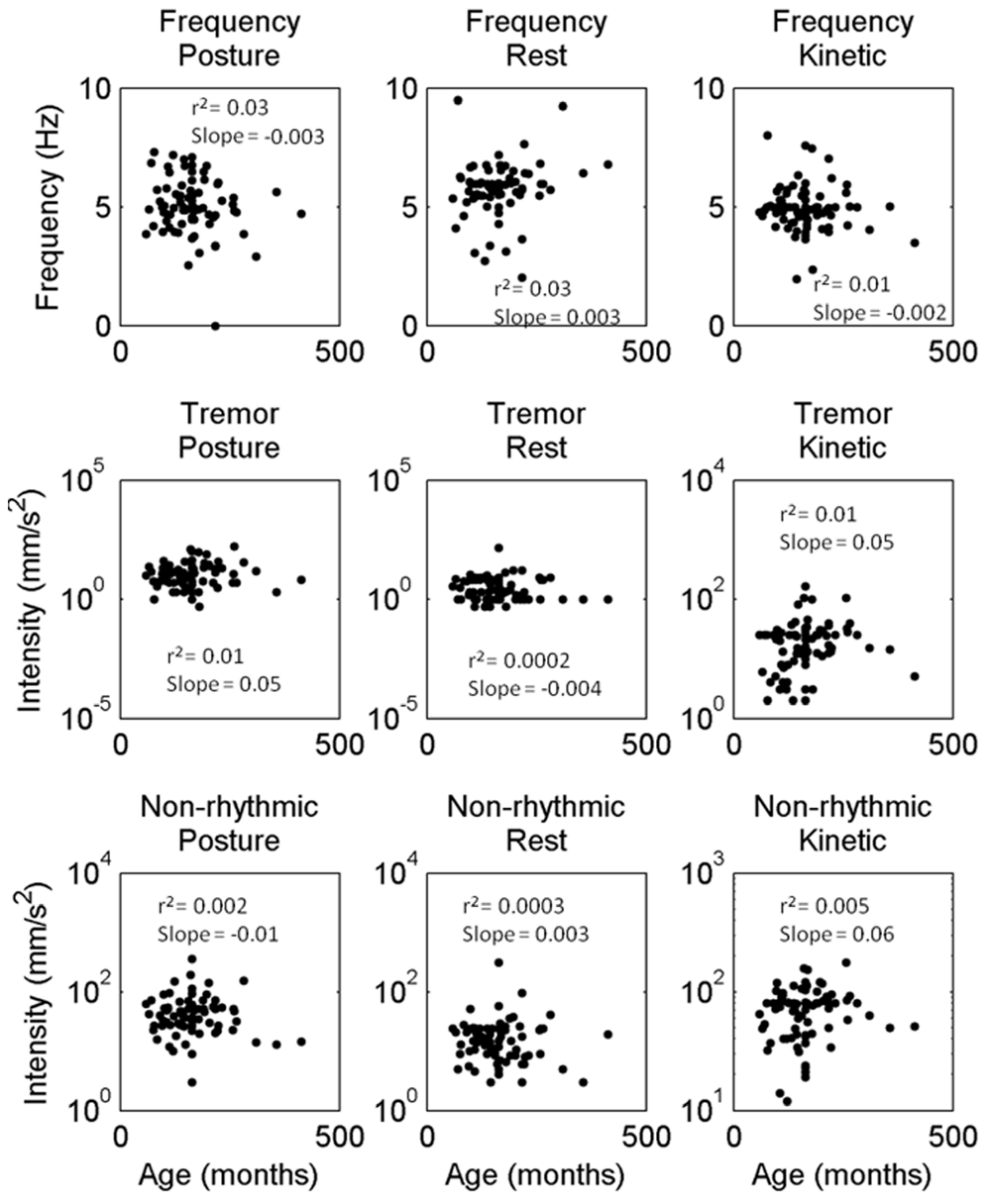
Correlation of frequency, tremor and non-rhythmic movement amplitude during postural, resting, and kinetic condition with subject’s age. Frequency, tremor and non-rhythmic movements during postural, resting, and kinetic conditions are plotted versus the subjects’ age in months. Slope and correlation coefficient by linear regression are depicted in the inset of each subplot. None of these parameters revealed any correlation.

In our cross-sectional study there was no relationship between the tremor frequency, tremor intensity, or non-tremor movement intensity and age in any of the three recording conditions. This lack of a relationship extended across a wide range of ages, including the pre-teen years of declining neurologic function and later years of relative stability (Figure 6). Weak values of r-squared coefficient in the inset of corresponding panel in Figure 6 quantified lack of relationship. The absence of such relationship suggests static (non-progressive) nature of hyperkinetic movement disorders in A–T subjects.

## Discussion

Progressive degeneration of the cerebellar Purkinje neurons is the hallmark neuropathology of A–T [11], [12]. Outside of the cerebellum there is no known consistent pathology, though some animal models of A–T suggest an abnormality of dopaminergic neurons within basal ganglia [13], [14]. The abnormal movements that we have identified in A–T are characterized by features classically associated with abnormalities in the cerebellum, but also by networks connecting cerebellum and the basal ganglia [15], [16], [17].

The abnormal movements characterized here include postural, kinetic, and resting tremors mixed with other non-rhythmic involuntary movements. There were two types of non-rhythmic movements: slow irregular movements appearing like athetosis or dystonic drifts, and relatively rapid non-rhythmic movements resembling dystonic jerks or myoclonus. Analysis of three dimensional accelerometer recordings permitted the separation of tremor from non-rhythmic rapid adventitious movements.

Resting tremor was generally of lower intensity and higher frequency compared to postural and kinetic tremor. The combination of features identified in subjects with A–T are similar to those described with disturbances of the cerebello-thalamic pathway [18] or as a mixed manifestation of basal ganglia and cerebellar involvement [19]. Neurophysiological studies in Parkinson’s disease have proposed that resting tremor is an oscillation in cerebello-thalamic-cortical circuits that are modulated by the basal ganglia [20], [21]. It was proposed that pathologies affecting dopaminergic neurons in the basal ganglia, such as Parkinson’s disease, unmask or modulate the oscillations of the cerebello-thalamic-cortical network [20]. It is therefore possible that the resting tremor in patients with A–T is due to abnormally increased oscillations in cerebello-thalamic-cortical circuits. Such oscillations can be hypothetically described by either known cerebellar deficits in A–T or speculated deficits in the basal ganglia.

Cases of genetically confirmed A–T have recently been described in which the dominant feature is dystonia [22], [23]. We also frequently observed slow, non-rhythmic athetosis or dystonic drifts in our cohort with A–T. Their pathophysiologic origin is less well localized, with proposed involvement of basal ganglia and cerebellum [24], [25]. The rapid non-rhythmic, and often high amplitude movements, were consistent with myoclonus or dystonic jerks. These movements were least apparent under resting conditions and were evoked more easily with muscle activation during the postural task, but were maximal during goal-directed movements of the kinetic task. This pattern of activation is characteristic of dystonic jerks [26]. Dystonia has been linked to deficits in the basal ganglia [27], [28], cerebellum [29], [30], [31], or disruption of the motor network between basal ganglia and cerebellum [25]. Alternatively (but non-exclusively), non-rhythmic rapid movements could be considered a form of action myoclonus. Similar movements have been identified in other neurodegenerative disorders of the cerebellum [32].

The neurodegeneration of A–T is complex, with many dimensions that manifest independence with respect to one another [33]. At issue for an understanding of underlying pathophysiology, and critical to clinical trial design, is an understanding of which and to what degree the independent elements of the disorder represent either a degenerative or a static developmental pathology. The observations in our cross-sectional study suggest the tremor and non-rhythmic adventitious movements of A–T are more static in nature, at least during the interval of ages studied here where assessment was possible. The results also suggest that accelerometry of hyperkinetic movement disorders in A–T could be a reliable parameter to measure the outcome of future pharmacological study.

## Supporting Information

Video S1 **Movie clip depicts disorders of upper-limb movement in one subject with A-T.**
(WMV)

## References

[1] Boder E, Sedgwick RP (1970) Ataxia-telangiectasia. (Clinical and immunological aspects). Psychiatr Neurol Med Psychol Beih 13–14: 8–16. 5006295

[2] Wells CE, Shy GM (1957) Progressive familial choreoathetosis with cutaneous telangiectasia. J Neurol Neurosurg Psychiatry 20: 98–104. 13429376 10.1136/jnnp.20.2.98 PMC497239

[3] Syllaba L, Henner K (1926) Ctribution a Lindependance de lathetose double idiopathique et congenitale. Revue Neurologique 5: 541–562.

[4] Cabana MD, Crawford TO, Winkelstein JA, Christensen JR, Lederman HM (1998) Consequences of the delayed diagnosis of ataxia-telangiectasia. Pediatrics 102: 98–100. 9651420 10.1542/peds.102.1.98

[5] Elble RJ (2005) Gravitational artifact in accelerometric measurements of tremor. Clin Neurophysiol 116: 1638–1643. 15905122 10.1016/j.clinph.2005.03.014

[6] Shaikh AG, Jinnah HA, Tripp RM, Optican LM, Ramat S, et al (2008) Irregularity distinguishes limb tremor in cervical dystonia from essential tremor. J Neurol Neurosurg Psychiatry 79: 187–189. 17872981 10.1136/jnnp.2007.131110 PMC2737356

[7] Shaikh AG, Crawford TO, Lederman HM, Tripp RM, Zee DS. A novel software algorithms for tremor analysis - comparing the novel approach with standard techniques; 2006; Banff, Alberta, Canada.

[8] Deuschl G, Raethjen J, Lindemann M, Krack P (2001) The pathophysiology of tremor. Muscle Nerve 24: 716–735. 11360255 10.1002/mus.1063

[9] Vaillancourt DE, Sturman MM, Verhagen Metman L, Bakay RA, Corcos DM (2003) Deep brain stimulation of the VIM thalamic nucleus modifies several features of essential tremor. Neurology 61: 919–925. 14557560 10.1212/01.wnl.0000086371.78447.d2

[10] Pincus SM (1991) Approximate entropy as a measure of system complexity. Proc Natl Acad Sci U S A 88: 2297–2301. 11607165 10.1073/pnas.88.6.2297 PMC51218

[11] Sedgwick RP, Boder E (1960) Progressive ataxia in childhood with particular reference to ataxia-telangiectasia. Neurology 10: 705–715. 14444443 10.1212/wnl.10.7.705

[12] Aguilar MJ, Kamoshita S, Landing BH, Boder E, Sedgwick RP (1968) Pathological observations in ataxia-telangiectasia. A report of five cases. J Neuropathol Exp Neurol 27: 659–676. 5687758

[13] Mount HT, Martel JC, Fluit P, Wu Y, Gallo-Hendrikx E, et al (2004) Progressive sensorimotor impairment is not associated with reduced dopamine and high energy phosphate donors in a model of ataxia-telangiectasia. J Neurochem 88: 1449–1454. 15009646 10.1046/j.1471-4159.2003.02278.x

[14] Eilam R, Peter Y, Elson A, Rotman G, Shiloh Y, et al (1998) Selective loss of dopaminergic nigro-striatal neurons in brains of Atm-deficient mice. Proc Natl Acad Sci U S A 95: 12653–12656. 9770541 10.1073/pnas.95.21.12653 PMC22886

[15] Shaikh AG, Marti S, Tarnutzer AA, Palla A, Crawford TO, et al (2009) Gaze fixation deficits and their implication in ataxia-telangiectasia. J Neurol Neurosurg Psychiatry 80: 858–864. 19357126 10.1136/jnnp.2008.170522

[16] Shaikh AG, Marti S, Tarnutzer AA, Palla A, Crawford TO, et al (2011) Ataxia telangiectasia: a "disease model" to understand the cerebellar control of vestibular reflexes. J Neurophysiol 105: 3034–3041. 21471399 10.1152/jn.00721.2010

[17] Lewis RF, Lederman HM, Crawford TO (1999) Ocular motor abnormalities in ataxia telangiectasia. Ann Neurol 46: 287–295. 10482258 10.1002/1531-8249(199909)46:3<287::aid-ana3>3.0.co;2-0

[18] Deuschl G (1999) Differential diagnosis of tremor. J Neural Transm Suppl 56: 211–220. 10.1007/978-3-7091-6360-3_14 10370914

[19] McAuley JH, Rothwell JC, Marsden CD, Findley LJ (1998) Electrophysiological aids in distinguishing organic from psychogenic tremor. Neurology 50: 1882–1884. 9633751 10.1212/wnl.50.6.1882

[20] Helmich RC, Janssen MJ, Oyen WJ, Bloem BR, Toni I (2011) Pallidal dysfunction drives a cerebellothalamic circuit into Parkinson tremor. Ann Neurol 69: 269–281. 21387372 10.1002/ana.22361

[21] Ni Z, Pinto AD, Lang AE, Chen R (2010) Involvement of the cerebellothalamocortical pathway in Parkinson disease. Ann Neurol 68: 816–824. 21194152 10.1002/ana.22221

[22] Worth PF, Srinivasan V, Smith A, Last JI, Wootton LL, et al.. (2012) Very mild presentation in adult with classical cellular phenotype of ataxia telangiectasia. Mov Disord. 10.1002/mds.25236 23143971

[23] Saunders-Pullman R, Raymond D, Stoessl AJ, Hobson D, Nakamura K, et al (2012) Variant ataxia-telangiectasia presenting as primary-appearing dystonia in Canadian Mennonites. Neurology 78: 649–657. 22345219 10.1212/WNL.0b013e3182494d51 PMC3286230

[24] LeDoux MS, Lorden JF (1998) Abnormal cerebellar output in the genetically dystonic rat. Adv Neurol 78: 63–78. 9750904

[25] Neychev VK, Fan X, Mitev VI, Hess EJ, Jinnah HA (2008) The basal ganglia and cerebellum interact in the expression of dystonic movement. Brain 131: 2499–2509. 18669484 10.1093/brain/awn168 PMC2724906

[26] Obeso JA, Rothwell JC, Lang AE, Marsden CD (1983) Myoclonic dystonia. Neurology 33: 825–830. 6683367 10.1212/wnl.33.7.825

[27] Tabbal SD, Mink JW, Antenor JA, Carl JL, Moerlein SM, et al (2006) 1-Methyl-4-phenyl-1,2,3,6-tetrahydropyridine-induced acute transient dystonia in monkeys associated with low striatal dopamine. Neuroscience 141: 1281–1287. 16766129 10.1016/j.neuroscience.2006.04.072

[28] Gernert M, Bennay M, Fedrowitz M, Rehders JH, Richter A (2002) Altered discharge pattern of basal ganglia output neurons in an animal model of idiopathic dystonia. J Neurosci 22: 7244–7253. 12177219 10.1523/JNEUROSCI.22-16-07244.2002 PMC6757886

[29] LeDoux MS, Lorden JF, Ervin JM (1993) Cerebellectomy eliminates the motor syndrome of the genetically dystonic rat. Exp Neurol 120: 302–310. 8491286 10.1006/exnr.1993.1064

[30] Campbell DB, North JB, Hess EJ (1999) Tottering mouse motor dysfunction is abolished on the Purkinje cell degeneration (pcd) mutant background. Exp Neurol 160: 268–278. 10630211 10.1006/exnr.1999.7171

[31] Pizoli CE, Jinnah HA, Billingsley ML, Hess EJ (2002) Abnormal cerebellar signaling induces dystonia in mice. J Neurosci 22: 7825–7833. 12196606 10.1523/JNEUROSCI.22-17-07825.2002 PMC6757989

[32] Shibasaki H, Thompson PD (2011) Milestones in myoclonus. Mov Disord 26: 1142–1148. 21626558 10.1002/mds.23673

[33] Crawford TO, Mandir AS, Lefton-Greif MA, Goodman SN, Goodman BK, et al (2000) Quantitative neurologic assessment of ataxia-telangiectasia. Neurology 54: 1505–1509. 10751267 10.1212/wnl.54.7.1505

